# Cryptochrome 2 extensively regulates transcription of the chloroplast genome in tomato

**DOI:** 10.1002/2211-5463.12082

**Published:** 2017-03-01

**Authors:** Paolo Facella, Fabrizio Carbone, Antonio Placido, Gaetano Perrotta

**Affiliations:** ^1^ ENEATrisaia Research CenterRotondella (MT)Italy; ^2^ Council for Agricultural Research and EconomicsThe Olive Growing and Olive Product Industry Research CentreRende (CS)Italy; ^3^ IBBE‐CNRBariItaly

**Keywords:** chloroplast, cryptochromes, light, *Solanum lycopersicum*, tiling array

## Abstract

Light plays a key role in the regulation of many physiological processes required for plant and chloroplast development. Plant cryptochromes (crys) play an important role in monitoring, capturing, and transmitting the light stimuli. In this study, we analyzed the effects of CRY2 overexpression on transcription of tomato chloroplast genome by a tiling array, containing about 90 000 overlapping probes (5‐nucleotide resolution). We profiled transcription in leaves of wild‐type and CRY2‐overexpressing plants grown in a diurnal cycle, to generate a comprehensive map of chloroplast transcription and to monitor potential specific modulations of the chloroplast transcriptome induced by the overexpression of CRY2. Our results demonstrate that CRY2 is a master gene of transcriptional regulation in the tomato chloroplast. In fact, it modulates the day/night mRNA abundance of about 58% of the 114 ORFs. The effect of CRY2 includes a differential extension of some transcripts at their 5′‐end, according to the period of the day. We observed that the influence of CRY2 on chloroplast transcription is not limited to coding RNA; a great number of putative noncoding micro RNA also showed differential accumulation pattern. To our knowledge, this is the first study that highlights how a photoreceptor affects the day/night transcription of the chloroplast genome.

**Abbreviations**
cp: chloroplast
CRY2‐OX: CRY2‐overexpressing plants
CRYs: cryptochromes
CV: coefficient of variance
FKF1: flavin‐binding kelch repeat F‐box1
LD: long‐day
LKP2: lov kelch protein 2
LRR: F‐box/leucine‐rich repeat protein
miRNA: noncoding micro RNA
NC: negative controls
ncRNA: noncoding RNA transcripts
NEP: nucleus‐encoded RNA polymerase
ORFs: open reading frames
PEP: plastid‐encoded RNA polymerase
PHOTs: phototropins
PHYs: phytochromes
rbcL: rubisco
RP: ribosomal protein
SIGs: sigma factors
TPR/PPR: tetra/pentatricopeptide repeat protein
UVR8: UV resistance locus 8
WGAs: whole‐genome tiling arrays
WT: wild‐type plants
ZTL: Zeitlupe
ZT: zeitgeber time

Light plays a key role in the regulation of many physiological processes required for plant and chloroplast (cp) development 1, 2. Light quality, quantity, periodicity, and duration are perceived by a set of different plant photoreceptors 3, 4. The blue/UV‐A/UV‐B photoreceptors include cryptochromes (CRYs), phototropins (PHOTs), UV resistance locus 8 (UVR8), and Zeitlupe family members (ZTL, FKF1, LKP2) 5, 6, 7, 8, 9, 10, while red/far‐red light photoreceptors include phytochromes (PHYs) 11, 12.

CRYs (CRY1, CRY2, and CRY3) are flavoproteins found in various taxa that are thought to have evolved from photolyases. In *Arabidopsis*, CRYs mediate light control of stem elongation, leaf expansion, photoperiodic flowering, and the circadian clock. CRY1 is the main blue light photoreceptor controlling the inhibition of hypocotyl elongation, anthocyanin accumulation, leaf and cotyledon expansion, extension growth, petiole elongation, gene expression, promotion of flowering, membrane depolarization, and phototropism 13, 14, 15, 16; CRY2 controls photoperiodic promotion of floral initiation 17, 18 and mediates the hypocotyls and internode shortening under both low‐ and high‐fluence blue light 19, 20; CRY3 is a CRY‐DASH protein that localizes to mitochondria and chloroplast 21 and is able to repair UV‐induced lesions in single‐stranded DNA 22 as well as in loop structures of double‐stranded DNA 23.

Cryptochromes can affect the transcription of some cp genes: CRY1 and CRY2 are involved in blue light‐specific coactivation of PSBD blue light‐responsive promoter 24, 25. The plastidial PSBD and PSBA genes encode the two chlorophyll‐binding proteins, D1 and D2, composing the reaction center core of photosystem II.

Chloroplast is a plant semiautonomous organelle whose genetic information is encoded in the nuclear and plastid genomes 25. It contains the cytoplasmic genetic system in close association with a complete photosynthetic apparatus. Chloroplast is also involved in several other aspects of plant cell metabolism, including biosynthesis of amino acids, lipids, vitamins, and pigments. The cp genome or plastome generally has a highly conserved organization composed of a single circular chromosome 120–200 Mbp long, with two large inverted repeats (IR), separated by the large and small single‐copy regions. The first complete plastome was sequenced from *Nicotiana tabacum* and *Marchantia polymorpha* 26, 27 and since then about 200 plastid genomes have been fully sequenced. The expression of a cp genome is finely regulated at the transcriptional, post‐transcriptional and post‐translational levels by complex regulatory patterns coordinated between nuclear and plastid compartments 28. It has been demonstrated that the transcription of cp genes responds to environmental and developmental cues 28. For example, sigma factors (sigs) are nuclear subunits of the plastid‐encoded RNA polymerase (PEP) and direct the initiation of promoter‐specific transcription by recognizing two consensus sequences of plastid gene promoters homologous to the −35 and −10 elements of *Escherichia coli* σ^70^‐type promoters 29. It has been established that PEP transcribes most of the photosynthesis‐related genes and plays a key role for cp development 30, 31. Moreover, a nucleus‐encoded RNA polymerase (NEP) is involved in the regulation of plastid transcription, adding a further layer of complexity to the cp RNA metabolism 32, 33. Genes encoding proteins required for housekeeping functions are often transcribed by NEP 34, but a significant number of genes hold promoters for both RNA polymerases and they can be transcribed either from PEP or NEP 34.

Furthermore, in recent years, many reported studies focused the overall structure and function of the cp genome as well as single cp genes through comparative transcription analyses 35, 36, 37, 38, 39, 40, 41, 42. DNA microarray technologies for cp transcriptomes have so far largely been applied to individual conditions and/or single mutations affecting cp functions 35, 36, 37, 40, 43. However, in contrast to in‐depth studies on nuclear gene expression, relatively little genome‐wide information is available regarding cp transcriptome fluctuations. An *Arabidopsis* oligonucleotide array containing more than 22 500 probe sets was used to identify novel *Arabidopsis* mutants impaired in cp gene expression and to elucidate interactive transcription networks 41, 44. Microarrays representing all cp genes for tobacco, potato, and tomato were also designed and produced 35, 40, 42.

Although microarray profiling designed on predicted features of a genome, such as intron–exon boundaries, coding regions, etc., have been applied to these studies, few truly whole‐genome tiling arrays (WGAs) have been designed to address these issues 45. Tiling arrays are useful for several purposes, and can be used to analyze both DNA and RNA content. They can also be used to discover transcribed genomic regions that are independent of previous annotations, to detect noncoding RNA transcripts (ncRNA) or to identify alternative RNA isoforms of known genes. This class of microarrays consists of partially overlapping probes that are tiled at regular intervals to cover the entire genome from end to end. This technology allows a more complete understanding of an organism's genomic organization, and should provide a dramatic improvement in the understanding of numerous biological processes.

Tomato (*Solanum lycopersicum*) has long served as a model system for plant genetics, development, pathology, and physiology, resulting in the accumulation of substantial information regarding the biology of this economically important crop. The sequencing of its nuclear and cp genome 46, 47 is complete. Four CRY genes have been discovered so far: two CRY1 (CRY1a and CRY1b), one CRY2 gene 48, 49 and one CRY3 50. The role of one of the CRY1 genes, CRY1a, has been elucidated through the use of antisense 51 and mutant 52 plants. CRY1a controls seedling photomorphogenesis, anthocyanin accumulation, and adult plant development. The overexpression of tomato CRY2 causes a high‐pigment phenotype, resulting in overproduction of anthocyanins and chlorophyll in leaves and of flavonoids and lycopene in fruits 53. Although recent microarray analyses evidenced as diurnal rhythms in gene expression are profoundly altered by CRY2 54, 55, also in response to hormone stimuli 56, there is not information about a possible CRY‐mediated regulation of cp transcription.

In this study, we analyzed the effects of CRY2 overexpression on transcription of tomato cp genome by a tiling array, containing about 90 000 overlapping cp probes (5‐nucleotide resolution). We profiled transcription in leaves of wild‐type (WT) and CRY2‐overexpressing (CRY2‐OX) plants grown in a diurnal cycle, to generate a comprehensive map of plastid transcription and to monitor potential‐specific modulations of cp transcriptome induced by the overexpression of CRY2.

## Materials and methods

### Plant material

WT and transgenic CRY2‐OX (line 52.3) 53 *Solanum lycopersicum* (cv. Moneymaker) plants were grown in a growth chamber for 28 days in long‐day (LD) conditions (14 h light – 25 °C/10 h dark – 23 °C). A light intensity of about 100 μmol^−2^·s^−1^ was provided by Osram (Munich, Germany) 11–860 daylight lamps.

To classify the time points at which the sampling was carried out, we used zeitgeber time (ZT) that is defined as the time in hours from the start of a normal day–light cycle 57. The green leaves of three plants for each genotype (WT and CRY2‐OX) were pooled at the following time points: ZT0 (dawn), ZT7 (midday), ZT14 (dusk), and ZT19 (midnight).

### Quantitative RT‐PCR

Total RNA (1 μg) from WT and CRY2‐OX plants, extracted as previously described 53, was reverse‐transcribed with oligo‐dT and Superscript III (Thermo Fisher Scientific, Waltham, MA, USA), according to the manufacturer's instructions. First strand cDNA (5 ng) was used as template for quantitative (Q) RT‐PCR. QRT‐PCR assays were carried out with gene‐specific primers, using an ABI PRISM 7900HT (Thermo Fisher Scientific) and the Platinum SYBR Green master mix (Thermo Fisher Scientific), according to the manufacturer's instructions. The primer sequences are: CRY2, GGGATCGTTTAATGCAAGCTATAATT and CGAGTTATCAAACACAACTTCAACAG; b‐actin, AGGTATTGTGTTGGACTCTGGTGAT and ACGGAGAATGGCATGTGGAA.

PCR conditions were: 5 min at 95 °C, followed by 45 cycles at 95 °C for 15 s, and at 58 °C for 60 s. At the end of the PCR, the thermocycler has been programmed to generate a thermal denaturation curve of the amplified DNA and to measure the melting temperature of the PCR product(s). The shape of the melting curve indicates whether the amplified products are homogeneous and the melting temperature provides confirmation that the correct product has been specifically amplified. Relative template abundance was quantified using the relative standard curve method described in the ABI PRISM 7900HT manual and the data were normalized for the quantity of the b‐actin transcript. Three PCR runs were carried out for each cDNA to serve as technical replicates and two independent experiments were carried out using two biological replicates for each genotype. Means from two independent experiments were subjected to SEM calculation, Student's *t* test using past software (http://folk.uio.no/ohammer/past/).

### Cp extraction and cp RNA purification

Cps were immediately extracted from leaves using the Chloroplast Isolation kit (Sigma‐Aldrich Co. LLC, St. Louis, MO, USA), according to the manufacturer's instructions for tobacco cp extraction.

Total nucleic acids extraction from purified cp samples was performed as described in Kahlau *et al*. 58 with minor modifications.

### Microarray analyses

Overlapping probes, designed over the tomato cp genome (NCBI: AM087200), were tiled at approximately 5‐base pair intervals as measured from the central position of adjacent oligonucleotides using Array Designer (Premier Biosoft, Palo Alto, CA, USA). Probes were synthesized *via in situ* on chips based on CustomArray^™^ semiconductor technology using a CustomArray^™^ Synthesizer (CustomArray Inc., Bothell, WA, USA). The resulting microarray contains over 90 thousand probes. Extra space on the microarray allowed us to replicate 67% of the probes, randomly chosen and 54 quality/negative controls (NC). The microarray design was deposited to the EBI public repository ArrayExpress (Accession number A‐MEXP‐2323).

For each experiment, 250 ng of DNA‐free cp RNA was reverse‐transcribed and amplified using TransPlex^®^ Complete Whole Transcriptome Amplification Kit (Sigma‐Aldrich Co. LLC) following the manufacturer's instructions. The amplified RNA was labeled in the presence of Cy5 using the ULS RNA ampULSe kit (Kreatech Diagnostics, Amsterdam, The Netherlands) following the manufacturer's instructions.

Three independent biological replicates were used for each point. All hybridizations showed a minimal Pearson correlation among biological replicates of 0.99 (*R* ≥ 0.99) and a mean coefficient of variance (CV) intrachip below 0.20.

All probes were grouped according to their GC content, ranging from 0 to 27. After this grouping, a specific component of GC‐dependent signal was observed. This effect was minimized by normalizing the probe signals onto the probe signal distribution of the most well‐represented GC content. GC normalization was performed using Office Excel 2010 (Microsoft corporation, Redmond, WA, USA) and the graphs were produced using past (http://folk.uio.no/ohammer/past/).

GC‐normalized values were, in turn, normalized with quantile‐based method using ProbeWeaver (CustomArray Inc.).

The signals produced for each array by negative controls were used to calculate the average level of background intensity and used to calculate the threshold intensity value (mean intensity of the negative controls plus 2 × standard deviation).

Arrays from each group (CRY2‐OX versus WT) were compared using a bioinformatic pipeline developed in the ‘An Integrated approach for the development of sustainable methods to control Tropical Theileriosis’ (http://www.theileria.org/ahdw/index.htm). The software suite includes a collection of interdependent scripts implemented in Perl, which generate and process a series of files using a variety of custom file formats. In particular, we used *sliding_window.pl* script that performs a function analogous to TAS (http://www.affymetrix.com/support/developer/downloads/TilingArrayTools/index.affx). We processed batch files (three replicates) for each conditions being tested. In total two types of comparison were carried out: (a) each experimental point versus its threshold intensity value in order to identify the detected probes; (b) each time point of CRY2‐OX versus its counterpart in WT to elucidate differential expression between the two genotypes. A two‐tailed ranked Wilcoxon test (unpaired) was used to compare a sliding window of probes in each of the two conditions. A bandwidth was set at 150. Detected regions were generated by interval analysis with a *P*‐value cutoff < 0.05 joining with a spacing of equal to or less than 3× step size and with a length equal or greater than 5× step size.

We followed the widely accepted Minimum Information About a Microarray Experiment (MIAME) guidelines for microarray analysis and verification 59 and microarray experiments have been deposited to the EBI public repository ArrayExpress (Accession number E‐MTAB‐1757).

## Results

**Figure 1:**
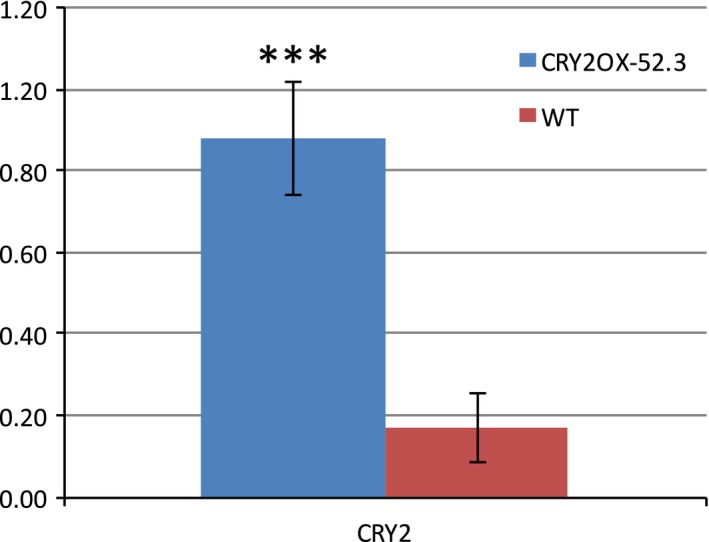
Expression of CRY2 gene in WT and CRY2‐OX (line 52.3) tomato plants analyzed by QRT‐PCR. Results are presented as a ratio after normalization with b‐actin. Data shown are the average of two biological replicates, with error bars representing SEM. ***Student's *t* test with *P* < 0.001.

We compared transcript levels of CRY2 via QRT‐PCR in CRY2‐OX (line 52.3) versus WT (Fig. 1). As expected, CRY2 was overexpressed about fivefold in the transgenic seedlings, confirming former results 53.

To appreciate the influence of CRY2 on the transcription of the tomato cp genome, we performed a large‐scale cp gene expression profiling comparing CRY2‐OX and WT plants.

High‐density tiling arrays containing 90k 35‐mer oligonucleotide probes were produced using CustomArray^™^ technology (CustomArray Inc.) and consisted of 30‐nucleotide overlapping probes, covering the entire cp genome (see Materials and methods). Total cp RNA extracted from WT and CRY2‐OX plants was random primed from three independent replicates per time point and used to hybridize the microarrays. To classify the time points at which the sampling was carried out, we used zeitgeber time (ZT) that is defined as the time in hours from the start of a normal day–light cycle 57. Tomato plants were grown under a light cycle of 14 h light/10 h darkness (LD) and sampled every 7 h from the presumptive dawn until dusk (ZT0, ZT7, and ZT14), and at 5 h after dusk (ZT19).

To calibrate the sequence‐specific probe effect, we used a process which involved three steps: (a) GC content normalization—based upon the correlation between probe signal intensity and its GC content; the probes were grouped by their GC content and, to adjust for differences in the dynamic ranges of the signals, the distributions were mapped to the distribution with the best representation (i.e., the set of probes with the same GC content with the highest population); (b) quantile normalization, where every slide was normalized to have the same cumulative frequency distribution; and (c) background correction and cutoff setting, where an error component of the intensities was estimated and eliminated; the signals produced by negative controls were used to calculate the average level of background intensity and then the threshold intensity value (mean intensity of the negative controls plus 2 × standard deviation).

In order to identify transcribed and differentially expressed segments, we applied one of the most widely used methods in tiling array expression analysis, introduced by Kampa *et al*. 60. In brief, the local expression levels of probes were estimated by calculating Hodge–Lehmann estimator over intensities of probes within genomic distance of bandwidth (150 nucleotides). Transcribed segments are collections of expressed probes, i.e. probes with a smoothed intensity above the given threshold. We also estimated the significance of differential expression using a Wilcoxon signed‐rank test (*P*‐value = 0.05). It tests for significant changes of probe intensities among states applied to local windows of given width centered around each probe. Data were visualized using the Integrated genome browser software (IGB, Affymetrix, Santa Clara, CA, USA).

### Transcripts affected by CRY2 overexpression

**Figure 2:**
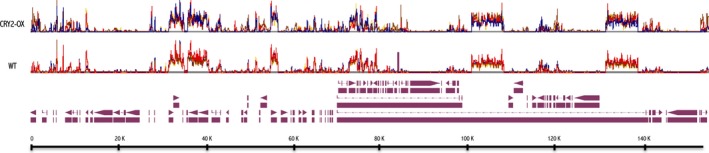
Visualization of tiling array intensity values along the whole tomato cp in WT and CRY2‐OX plants. The plot shows the normalized and subtracted background hybridization intensities (*y* axis) along the cp genome (*x* axis) of each genotype for all time points (zeitgeber times, [ZT]). ZT0: pink, ZT7: yellow, ZT14: orange, ZT19: blue. Annotated ORFs are shown as arrow boxes.

cp mRNA levels between CRY2‐OX and WT plants were compared during a light/dark cycle (14 h of light/10 h of dark) at the four time points described above (Fig. 2).

**Figure 3:**
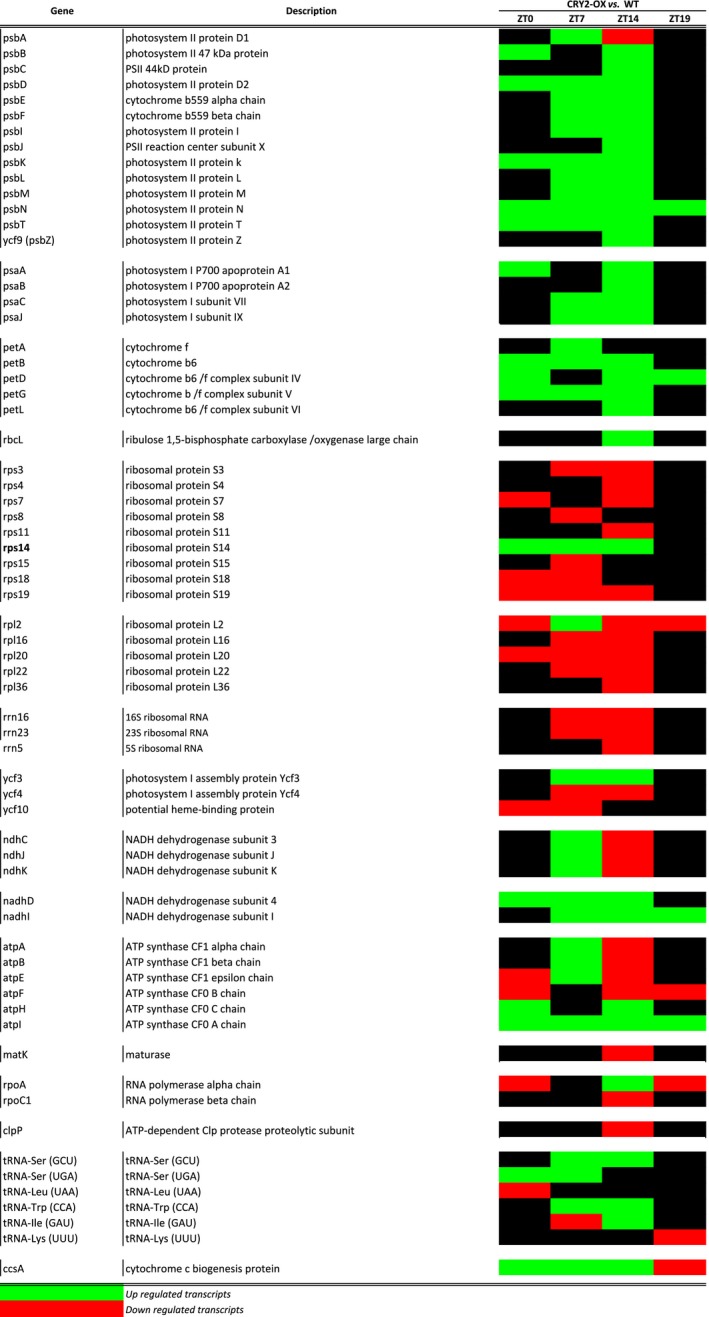
Transcripts differentially regulated between CRY2‐OX and WT plants during a day/night cycle. Red and green boxes indicate respectively down‐ and upregulation of the genes in CRY2‐OX plants with respect to WT.

Sixty‐six transcripts were identified as differentially expressed in CR2‐OX versus WT tomatoes in at least one of the time points analyzed, 58% of cp ORFs (Fig. 3), indicating a considerable impact of CRY2 on the whole cp transcription apparatus. This dramatic effect appears to be light‐dependent, as the majority of CRY2‐induced alterations occurred at specific day time: 66% at ZT7 and 87% at ZT14 (Fig. 3). During the presumptive night (ZT19), only nine transcripts are affected by the overexpression of CRY2, (Fig. 3); hence, the effect of CRY2 overexpression on tomato cp transcriptome appears to be amplified during the diurnal phase of the day and minimized during the night and around the presumptive dawn.

**Figure 4:**
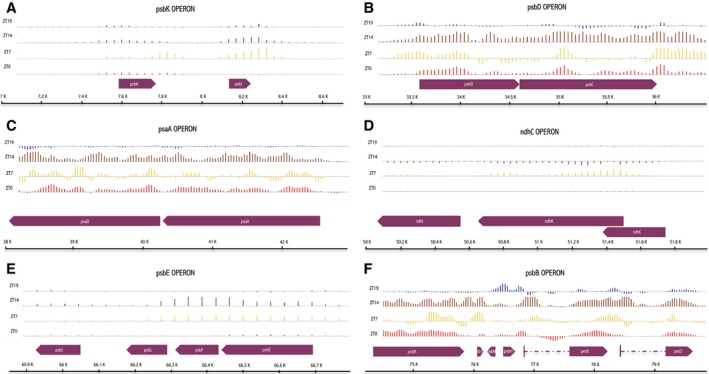
Differentially expressed genes of selected cp operons. The plot shows the ratio of the normalized and subtracted background hybridization intensities measured for CRY2‐OX to WT (*y* axis) along the cp genome (*x* axis) of psbK, psbD, psaA, ndhC, psbE, and psbB operons (A‐F) for each time point (zeitgeber times, [ZT]). ORFs are shown as arrow boxes.

**Figure 5:**
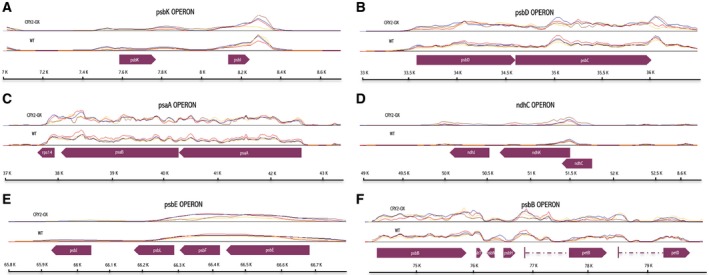
Visualization of tiling array intensity values along cp operons in WT and CRY2‐OX plants. The plot shows the normalized and subtracted background hybridization intensities (*y* axis) along psbK, psbD, psaA, ndhC, psbE, and psbB operons (A–F) (*x* axis) of genotype for all time points (zeitgeber times, [ZT]). ZT0: pink, ZT7: yellow, ZT14: orange, ZT19: blue. ORFs are shown as arrow boxes.

Among the 66 transcripts regulated in transgenic tomatoes, we found the great majority of the cp photosynthesis‐related genes; 88% of the total number of the transcripts encoding photosystem I and II proteins (Figs 3 and 4). Excepting petA and psbA, all these genes demonstrated a robust upregulation trend at the presumptive dusk (ZT14 – 21 genes out of 23); furthermore, we also found widespread upregulation at ZT7 (15 genes out of 23) (Figs 3 and 4). PsbN and petD transcripts were the only photosynthetic genes still upregulated during the night (ZT 19 – Figs 3 and 4). As expected, the photosystem‐related genes clustered in operons exhibited very similar patterns of regulation (Fig. 5). Finally, also the large subunit of RuBISCO (rbcL) transcripts were upregulated in CRY2‐OX at ZT14 (Fig. 3).

All these data show a synchronized signal of upregulation for the structural cp photosystem genes mediated by cryptochrome 2: further analysis are required to investigate whether such upregulation may result in improvement of the photosynthetic machinery in CRY2‐OX tomatoes.

On the other hand, most of the transcripts involved in translation of cp‐encoded genes were downregulated in CR2‐OX, showing an opposite pattern of modulation with respect to photosynthetic genes. Remarkably, 13 ribosomal protein (rp) encoding transcripts (62% of cp rp ORFs) exhibited downregulation of their mRNA in CRY2‐OX in at least one time point (Fig. 3). The sole exception to this trend is represented by the rp S14 (rps14) transcripts, upregulated from ZT0 to ZT14 in transgenic tomatoes (Fig. 3). In tobacco, the rps14 gene is transcribed as a part of the psaA operon, which includes two photosystem I genes, psaA and psaB 34. Our data showed a similar organization in tomato cp genome; hence rps14 upregulation could be the result of a transcription carryover of the adjacent photosystem genes to be corrected at a post‐transcriptional level.

Accordingly, transcripts for ribosomal RNA genes rrn5, 16, and 23 were downregulated in CRY2‐OX at ZT7 and ZT14, suggesting the occurrence of a CRY2‐mediated general signal of repression of the cp genetic system (Fig. 3).

A number of other ORFs coding for ATP synthase, NADH dehydrogenase, and tRNA did not exhibit a homogeneous transcript alteration pattern along the day, hinting a possible time‐specific alteration triggered by CRY2 (Fig. 3).

Our study reveals that CRY2 has a massive effect on the transcriptional cp apparatus of tomato, increasing the mRNA quantities of the photosynthetic genes and, at the same time, decreasing those of the genes coding for rp and ribosomal RNA. Thus, modulating transcription levels of more than 50% of cp ORFs, cryptochrome 2 is a plausible candidate master gene for regulation of the cp transcription machinery in tomato.

### Mapping transcript initiation and promoter motives

**Figure 6:**
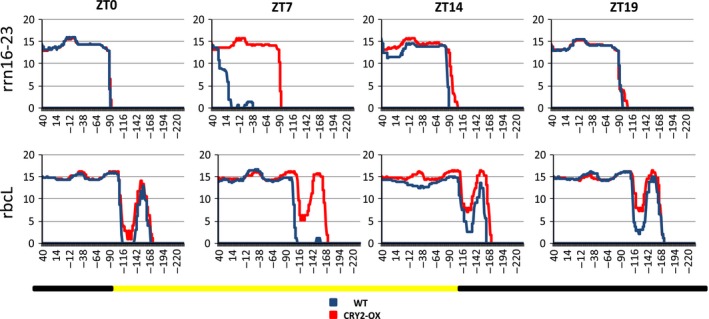
Visualization of putative transcriptional start sites of rbcL and 16‐23 rrn genes in CRY2‐OX and WT plants during a day/night cycle. The plot shows hybridization intensities (*y* axis) along the region upstream of start codon (ATG) of rbcL and 16‐23 rrn operons (*x* axis in bp). Data of all time points (zeitgeber times, [ZT]) are shown per genotype. Yellow and black bars along the horizontal axis represent light and dark periods, respectively.

As already described above, the effect of CRY2 overexpression on cp transcription appears to be largely light‐dependent, as the most relevant changes on the quantity of plastidial RNA occur during the light phase of the day (ZT7 and ZT14). Starting from these results, we examined, in CRY2‐OX and WT, each transcribed segment along the cp genome to map transcription initiation points. To this respect, no significant differences were observed for most genes neither between the two genotypes nor within a single genotype at different time points (data not shown). Noteworthy, exceptions concern rbcL and rrn16‐23 genes that showed a different transcription start site at ZT7 in WT plants with respect to all the other time points analyzed in both the genotypes (Fig. 6). They presented a shift of the transcription start, closer to their ATG, giving rise to shorter mRNA (Fig. 6). Our results clearly indicate that the transcription start of some cp genes and consequently, the length of their mRNA are influenced by two factors: the period of the day and the quantity of CRY2 protein. In fact, in CRY2‐OX at ZT7, no alterations of the transcription start site were noticed, demonstrating a resetting effect of the CRY2 overexpression on this modification. However, we cannot rule out that the observed differences are the result of CRY2 induced post‐transcriptional processing rather than the effect of alternative transcription initiation sites.

Having observed an opposed trend of RNA accumulation in CRY2‐OX versus WT plants, downregulation for the cp genetic system genes and upregulation for the photosynthesis‐related genes, we wondered whether these diverging effects were possibly related to the distinct transcription machineries of chloroplast, the plastid‐encoded *E. coli‐like* RNA polymerase, PEP, and the nucleus‐encoded bacteriophage‐type RNA polymerase, NEP 32. To this end, we scanned the transcription activity in regions upstream of the start codon (ATG) of each expressed gene, in order to map active promoters for each transcript. It was not possible to identify a unique active promoter for most of the genes, because of the overlapping of PEP and NEP promoter *consensus*. Besides, the structure of NEP promoters is very variable and, in many cases, elusive 61. Three different types of NEP promoters have been identified in plants, so far: class Ia promoters, characterized by an highly conserved YRTa core motif immediately upstream of the transcription initiation site 62; class Ib promoters, which present a GAA‐box further upstream of the YRTa‐box 63; class II promoters, which lack the YRTa‐motif and differ completely from the class I ones, presenting a specific −5 to +25 sequence able to support NEP transcription initiation 64.

**Figure 7:**
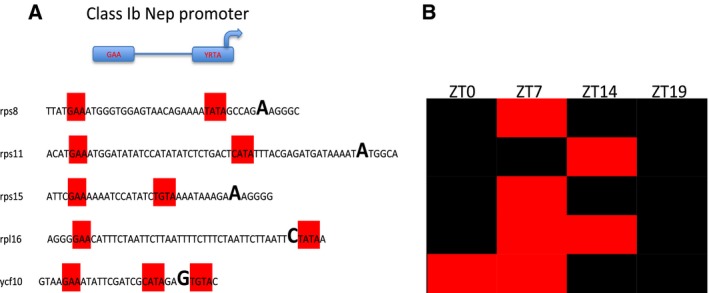
cp genes showing active NEP promoters. (A) Sequences of active class Ib NEP promoters. The GAA and YRTa motifs are included in red boxes. The first transcribed nucleotide is bolded. (B) Expression of the selected genes in CRY2‐OX versus WT plants during a day/night cycle. Red and green boxes indicate, respectively, down‐ and upregulation.

We decided to consider class Ib promoter genes because they have a more definite structure (GAA‐box and YRTa‐motif). Interestingly, five downregulated genes showed NEP class Ib sequences (Fig. 7). All but one of these transcripts code for ribosomal proteins. We also found one class Ib promoter upstream of the transcription start of ycf10 gene that seems to have a role in the inorganic carbon uptake in chloroplast 65.

**Figure 8:**
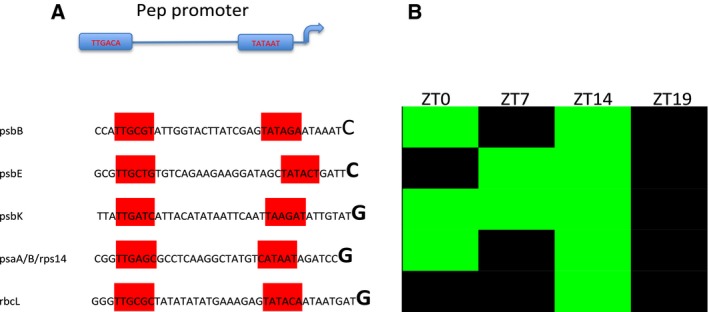
cp genes showing active PEP promoters. (A) Sequences of active PEP promoters. The TTGACA‐ and TATAAT‐like motifs are included in red boxes. The first transcribed nucleotide is bolded. (B) Expression of the selected genes in CRY2‐OX versus WT plants during a day/night cycle. Red and green boxes indicate, respectively, down‐ and upregulation.

The structure of PEP promoters was deeply studied in mono‐ and dicotyledon plants, as well 33. A typical PEP promoter contains a variant of the −10 (TATAAT) and −35 (TTGACA) consensus sequences of canonical σ^70^‐type *E. coli* promoters 66, 67. It is well known that PEP polymerase is mainly involved into the transcription of photosynthetic genes and it seems to be light regulated 68. Consequently, we analyzed the promoter structures of photosynthesis‐related genes, upregulated in CRY2‐OX. A number of these genes were unambiguously transcribed from a PEP promoter: psbB, psbE, psbK, psaA, and rbcL (Fig. 8), confirming the preference of PEP in transcribing photosynthesis genes in tomato, as well.

Although our experiments do not draw a full picture of the CRY2 effect on the cp transcription machinery, they give rise to some interesting interpretations. We can speculate that CRY2 is able to modulate the relative activity of both cp polymerases, PEP and NEP, stimulating the activity of the first one and, at the same time, reducing the action of the second one. The overall effect of this dual regulation is the increase of the photosynthetic‐related transcripts at the expense of the genetic system‐related ones.

### Plastidial miRNA regulated by CRY2

**Figure 9:**
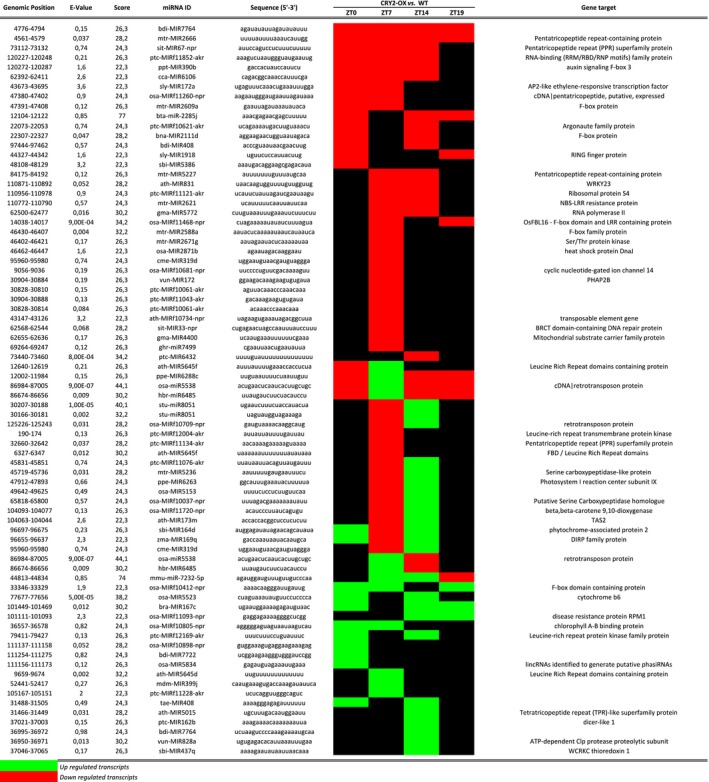
miRNA differentially regulated between CRY2‐OX and WT plants during a day/night cycle. Red and green boxes indicate, respectively, down‐ and upregulation of the miRNA in CRY2‐OX plants with respect to WT.

**Figure 10:**
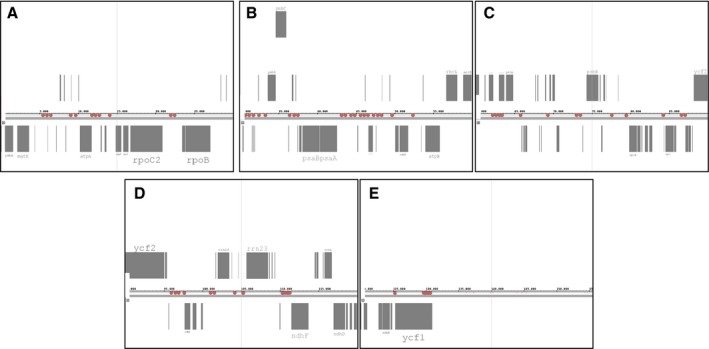
Location of miRNA along the tomato cp genome. The whole tomato genome was divided into five subregions (A–E). Putative miRNA are represented by colored circles. For gene acronyms, see text. ORFs are shown as gray boxes.

We analyzed the global transcription of tomato cp genome in order to isolate noncoding micro RNA (miRNA) showing different pattern of expression in CRY2‐OX versus WT plants. All transcribed segments were submitted to the miRBase search tool 69 and then blasted against the plant noncoding RNA database (http://structuralbiology.cau.edu.cn/PNRD/). A total of 79 cp miRNA were identified in WT plants and all of them appeared to be differentially expressed in CRY2‐OX tomatoes (Fig. 9). CRY2‐modulated miRNA were not randomly distributed in the tomato chloroplast transcriptome, and they were concentrated in noncoding regions (58 of 79): 40 miRNA were in intergenic regions and 18 within introns (Fig. 10).

As already observed for cp genes, the majority of CRY2‐modulated miRNA were altered during the light phase of the day (75% at ZT 7 and 57% at ZT 14) (Fig. 9). However, during the presumptive night (ZT19), only 12 miRNA were significantly changed (Fig. 9). This expression trends underline a CRY2‐induced time correlation in both coding and noncoding cp RNA accumulation. Furthermore, we analyzed the putative gene targets of the 76 miRNA, using a plant noncoding RNA database‐specific tool (http://structuralbiology.cau.edu.cn/PNRD/targets_search.php). Interestingly, we found six miRNA having tetra/pentatricopeptide repeat (TPR/PPR) proteins as putative targets (Fig. 9). This family of proteins plays a role in stabilization of specific cpRNA: in fact, it has been shown that the association of PRRs with processed transcripts protects them from RNAse attack 70, 71. Besides, 13 of the 76 CRY2‐regulated miRNA share F‐box/leucine‐rich repeat (LRR) genes, involved in the plant response to pathogen infections 72, as putative targets (Fig. 9).

## Discussion

We used a genome‐wide approach, by tiling array transcription profiling, to define the effect of CRY2 on tomato cp transcript accumulation. Tomato cp is a simple and relatively small genome that enabled to set up a 5‐nucleotide resolution tiling array providing opportunities to rapidly characterize novel transcript features. In contrast to traditional microarrays, which contain a number of probes with same thermodynamic characteristics, tiling arrays include probes overlapping along all the DNA sequence, providing a continuous hybridization signal.

To our knowledge, only 10 cp genome‐specific microarrays have been developed, and almost all of them cover only coding regions [35, 36, 37, 38, 39, 40, 42, 45, 73, 74, 75, 76]. Our microarray tiles the whole tomato cp genome with a much higher resolution than any cp array reported so far and enables to reveal gene transcript fluctuations, miRNA, alternative promoter usage, and identification of sites of transcript initiation.

Our results demonstrate that CRY2 is a master gene of the transcriptional regulation in tomato chloroplast. In fact, it modulates the daily mRNA abundance of about 58% of the 114 cp ORFs (Fig. 3). This dramatic effect is influenced by the period of the day and, consequently, by the presence of light. Indeed, the strongest CRY2‐induced perturbation of cp transcripts occurs during the light phase of the day (ZT7 and ZT14). It means that the role of the light is epistatic with respect to the overexpression of CRY2; that, alone, is not able to cause significant transcriptional changes during the presumptive night (ZT19 and ZT0). Hence, despite the massive perturbation caused by overexpression of CRY2, the tomato system is still able to correctly recognize light and dark phases, suggesting that other molecular factors (probably other photoreceptors) participate in the light/dark transcriptional modulation of the chloroplast genome 77, 78.

The effect of CRY2 on the chloroplast transcriptome is not limited to the mRNA quantity of cp coding regions, and it also includes a differential extension of some transcripts at their 5′‐end, according to the period of the day. In fact, we found two genes, rbcL and rrn16‐23, presenting an apparent modification of the transcriptional start site during the light phase of the day, ZT7, with respect to the other time points analyzed; this modification was present only in WT plants, whereas it was absent in transgenic tomatoes.

This remarkable finding makes it evident that the activity of tomato cp transcriptional apparatus is influenced by the period of the day and, more important, this influence is CRY2‐mediated. Indeed, in CRY2‐overexpressing plants, the alteration of the 5′‐ transcript length at ZT7 was totally reset, indicating that changes in the amount of CRY2 protein could drive the positioning of the cp RNA polymerases or, alternatively, the post‐transcriptional processing of RNA.

One of the most interesting results is that CRY2 upregulates almost all the photosynthesis‐related genes (88%) and, at the same time, downregulates a large number of the cp genetic system genes (Fig. 3). In angiosperm, cp transcription is mediated by two distinct types of RNA polymerases, PEP and NEP; PEP is the predominant polymerase for transcription of photosynthesis machinery genes, while NEP is required for transcription of housekeeping genes such as ribosomal proteins 32, 34. To investigate the relative role of the two polymerases in tomato cp transcription, we mapped the transcription upstream of the start codon (ATG) of each expressed ORF. We found that a consistent number of CRY2‐OX versus WT upregulated photosynthesis‐ related transcripts were unambiguously transcribed from a PEP promoter: psbB, psbE, psbK, psaA, and rbcL (Fig. 8). Conversely, among downregulated genes, we found at least five genes transcribed by NEP (class Ib promoter) (Fig. 7). One possible hypothesis is that CRY2 stimulates PEP while repressing NEP possibly incrementing the photosynthesis‐related proteins in tomato chloroplasts. This is supported by previous experiments by Giuliano and collaborators 53, which showed that tomato CRY2‐OX plants accumulate chlorophylls and carotenoids in leaves. It is well known that rates of PEP transcription are higher in the light rather than in the dark and that its activity/specificity is regulated by nuclear encoded sigma‐like transcription factors (SIGs) 68, 79. In *Arabidopsis*, six SIGs have been identified 80, 81, 82 and two of them, AtSIG1 and AtSIG5, are light‐induced at the transcription level 83. Red light on dark‐adapted plants strongly induces AtSIG1 transcripts, while blue light causes rapid accumulation of AtSIG1 and AtSIG5 transcripts. Moreover, Onda *et al*. 83 showed that AtSIG5 induction is caused by CRY2 at low fluences of light. Starting from those studies, we inferred that in cp tomatoes, the increase of PEP promoter activity was possibly related to a CRY2 direct regulation of SIG transcripts, as it occurs in *Arabidopsis*. Therefore, we compared the expression of six tomato SIG genes between CRY2‐OX and WT plants by QRT‐PCR, during a 24‐h cycle (LD conditions). Surprisingly, we did not find significant differences of SIGs gene expression between the two genotypes (data not shown). It must be considered that the stabilization/activation of the PEP is a multifaceted process in which, aside from the sigma factors, a plethora of other factors such as pTAC and PPR proteins (see below) are involved. Furthermore, other than light, a number of internal and external signals have an influence on PEP‐induced cp transcription, like redox status, protein phosphorylation, and heat stress 84. Therefore, in principle, tomato CRY2 could promote PEP activity independently by SIGs. However, to address the question about a possible CRY2‐mediated modulation of PEP and NEP, further analyses are required.

We found a large number (76) of miRNA whose accumulation is modulated by overexpression of CRY2 (Fig. 9) suggesting that CRY2 influence on the cp transcription is not limited to coding RNA.

miRNA CRY2‐mediated regulation appears to be strictly light‐dependent as already observed for cp ORFs. Given the large number of altered miRNA, it is conceivable that CRY2 controls post‐transcriptionally some cp genes through specific miRNA. To support this hypothesis, we found that a number of them share homology with *Arabidopsis* miRNA that target TPR/PPR proteins. TPR/PPR proteins stabilize their target mRNA protecting them against exonucleases, using miRNA as footprints 71. Additionally, many of the most frequent phenotypes associated with mutations in PPR genes result in deficits in energy supply, caused by defects in photosynthesis 85. Therefore, CRY2 might regulate the transcription of cp genes acting directly on the transcription of the target genes, possibly either by modulating PEP and NEP activity, either influencing RNA stability through activation/inhibition of specific PPR‐targeted miRNA.

Among CRY2OX versus WT differentially regulated cp miRNA, we also found 13 sequences presenting F‐box/LRR genes as silencing targets. LRR proteins belong to the large group of resistance genes, involved in the plant innate immune system that recognizes specific pathogen infection and triggers resistance responses 72. Recently, it has been demonstrated in many plant species including tomato that a number of LRR transcripts are regulated by nuclear miRNA, catalyzing cleavage and silencing of LRR transcripts 86, 87, 88.

In our view, the presented results provide new insights into a pivotal role of CRY2 in the transcriptional and post‐transcriptional regulation of cp transcripts; such a wide regulation could deeply affect some fundamental physiological plant processes like photosynthesis and defense responses.

## Author contributions

PF analyzed the data and wrote the manuscript. FC prepared the experiments, performed data analyses and wrote the manuscript. AP prepared the experiments. GP wrote and reviewed the manuscript.
